# Human Glycolipid Transfer Protein (GLTP) Expression Modulates Cell Shape

**DOI:** 10.1371/journal.pone.0019990

**Published:** 2011-05-18

**Authors:** Yongguang Gao, Taeowan Chung, Xianqiong Zou, Helen M. Pike, Rhoderick E. Brown

**Affiliations:** 1 The Hormel Institute, University of Minnesota, Austin, Minnesota, United States of America; 2 School of Biotechnology, Yeungnam University, Kyeongsan, Republic of Korea; Faculdade de Medicina, Universidade de São Paulo, Brazil

## Abstract

Glycolipid transfer protein (GLTP) accelerates glycosphingolipid (GSL) intermembrane transfer via a unique lipid transfer/binding fold (GLTP-fold) that defines the GLTP superfamily and is the prototype for GLTP-like domains in larger proteins, i.e. phosphoinositol 4-phosphate adaptor protein-2 (FAPP2). Although GLTP-folds are known to play roles in the nonvesicular intracellular trafficking of glycolipids, their ability to alter cell phenotype remains unexplored. In the present study, overexpression of human glycolipid transfer protein (GLTP) was found to dramatically alter cell phenotype, with cells becoming round between 24 and 48 h after transfection. By 48 h post transfection, ∼70% conversion to the markedly round shape was evident in HeLa and HEK-293 cells, but not in A549 cells. In contrast, overexpression of W96A-GLTP, a liganding-site point mutant with abrogated ability to transfer glycolipid, did not alter cell shape. The round adherent cells exhibited diminished motility in wound healing assays and an inability to endocytose cholera toxin but remained viable and showed little increase in apoptosis as assessed by poly(ADP-ribose) polymerase cleavage. A round cell phenotype also was induced by overexpression of FAPP2, which binds/transfers glycolipid via its C-terminal GLTP-like fold, but not by a plant GLTP ortholog (ACD11), which is incapable of glycolipid binding/transfer. Screening for human protein partners of GLTP by yeast two hybrid screening and by immuno-pulldown analyses revealed regulation of the GLTP-induced cell rounding response by interaction with δ-catenin. Remarkably, while δ-catenin overexpression alone induced dendritic outgrowths, coexpression of GLTP along with δ-catenin accelerated transition to the rounded phenotype. The findings represent the first known phenotypic changes triggered by GLTP overexpression and regulated by direct interaction with a p120-catenin protein family member.

## Introduction

Glycosphingolipids (GSLs) are key components of eukaryotic cellular membranes where they play important roles in surface adhesion, neuroregeneration, differentiation, drug resistance, and apoptosis [1]–[7]. In cells, GSL trafficking to various destinations occurs by both vesicular and nonvesicular mechanisms [7]–[12]. The latter mechanism involves a protein fold known as the glycolipid transfer protein (GLTP)-fold [9], [13]. The GLTP-fold utilizes a unique all α-helical conformation, arranged in a two-layer ‘sandwich motif’ that includes a single glycolipid binding site [13]–[15]. In contrast, lipid binding/transfer protein folds that bind other lipids are dominated by β-sheet, i.e. β-grooves/concave cups and β-barrels, or helical bundles stabilized by multiple disulfide-bridges, i.e. saposin-folds [9], [13], [14]. The GLTP-fold also represents a novel peripheral amphitropic motif with respect to membrane interaction [9], [12], [16]. Together, these distinct features have established the human GLTP-fold as the prototype of the new GLTP superfamily [9].

In human cells, two GLTP-folds are known to function in nonvesicular trafficking of GSLs: *i)* GLTP, a small soluble protein (209 a.a.) encoded by *GLTP* (chromosome 12; locus 12q24.11); *ii)* phosphatidylinositol 4-phosphate adaptor protein-2 (FAPP2; 519 a.a.) containing a C-terminal GLTP-fold encoded by *PLEKHA8* (chromosome 7; locus 7p21-p11.2) [9], [10], [12], [17], [18]. The issue of whether defective expression or mutation of GLTP-folds can be manifested as cell phenotype alterations has remained unexplored despite the established roles that GSLs are known to play in cellular adhesive interactions [1], [2], [19], [20]. In the present study, we have evaluated whether severe change in human *GLTP* mRNA expression alters cell morphology and whether proteins known to be involved in cell spreading and adhesion interact with GLTP in functionally significant ways.

## Results

### Dramatic changes in cell shape are induced by GLTP overexpression, but not by RNAi-mediated GLTP knockdown

**Figure 1:**
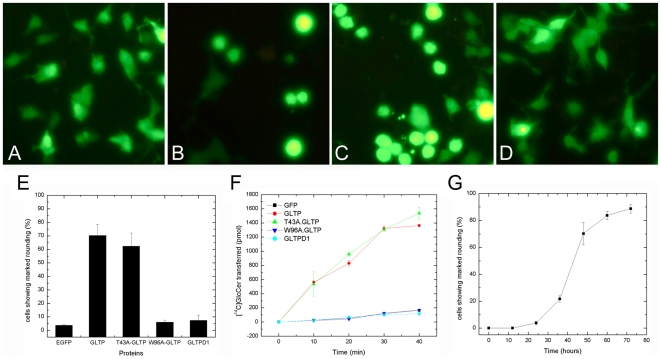
GLTP overexpression induces changes in cell morphology. **A**) HeLa cells overexpressing GFP; **B**) EGFP-GLTP, **C**) GFP-T43A-GLTP, and **D**) GFP-W96A-GLTP were analyzed by fluorescence microscopy 48 h after transfection. The overexpression of wtGLTP or T43A-GLTP induced dramatic rounding of HeLa cells. Overexpression of W96A-GLTP, a mutant unable to bind/transfer glycolipid, failed to induce cell rounding. **E**) Percentage of cells showing the rounded phenotype at 48 h after transfection. Data presented in the bar graph are the average of at least three independent experiments. **F**) Time-dependence for induction of the rounded phenotype in HeLa cells after transfection. Cell transfection efficiency was ∼60%. **G**) Glycolipid intermembrane transfer activities of HeLa cells overexpressing wtGLTP, T43A-GLTP, and W96A-GLTP.

To determine whether overexpression of human GLTP can alter cell phenotype, GLTP under the control of cytomegalovirus (CMV) promoter was overexpressed in HeLa cells while monitoring cell morphology (Fig. 1). Up to 24 h post transfection, the shape of the adherent cells remained almost completely unaffected. However, by 48 h post transfection, the cell phenotype had dramatically changed with ∼70% of the transfected adherent cells showing a markedly rounded shape (Fig. 1B, 1E, & 1F). GFP-GLTP expressed in the rounded cells was fully capable of transferring glycolipids (Fig. 1G). A similar outcome was observed for cells expressing T43A-GLTP (Fig. 1C & 1E), which contained a benign mutation adjacent to the glycolipid liganding site (Fig S1) that did not interfere with glycolipid transfer activity (Fig. 1G). Notably, however, the rounded cell morphology was not induced in cells overexpressing GFP-W96A-GLTP, a site mutation that blocks glycolipid binding and transfer [13] (Fig. 1D, 1E, & 1G). The bulk of cells overexpressing GFP-W96A-GLTP maintained their normal, elongated, and somewhat irregular shape, characteristics also shared by mock-transfected cells expressing GFP alone (Figs. 1A & 1E). The rounded-cell phenotype also was observed in HEK-293 cells overexpressing GLTP, but not in A549 cells by 72 h (Fig. S2).

**Figure 2:**
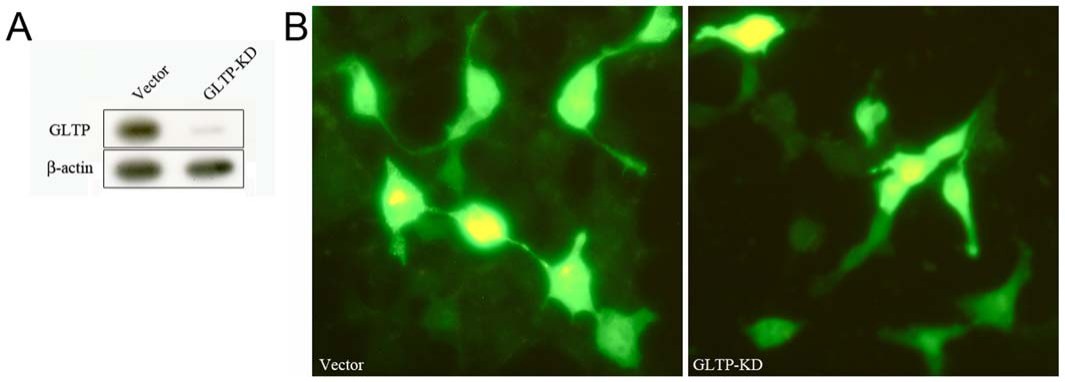
*GLTP* knockdown by RNAi does not alter cell phenotype. **A**) HeLa cells transfected with empty vector (control) or RNAi to *GLTP* mRNA (pSuper.puro-egfp-214) were analyzed by Western blot using β-actin expression as positive control. **B**) Epifluorescence microscopy showed that HeLa cell shape was not altered when *GLTP* expression was knocked down by siRNA.

Because the preceding findings indicated that high level expression of active GLTP induces cell phenotypic change, we also determined whether siRNA-induced inhibition of endogenous *GLTP* expression alters cell morphology. The high efficiency of the *GLTP* RNAi knock-down pSuper.retro construct used in our experiments is shown in Figure 2A. Despite efficient *GLTP* knock-down, HeLa cell shape remained normal and unaffected (Fig. 2B).

### Does GLTP-induced transition to a round phenotype affect cell dynamics?

**Figure 3:**
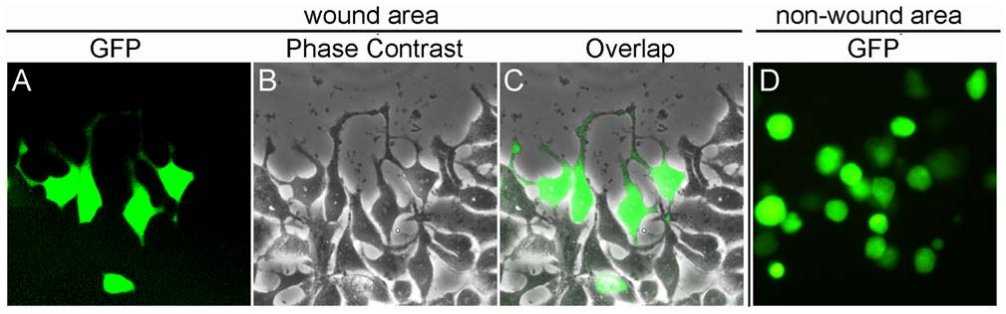
Rounded HeLa cells overexpressing GLTP exhibit diminished mobility. **A–C**) Wound closure assays showed that normal shaped cells are able to migrate longer distances, as indicated by their frequent detection in the centre of the wound. **D**) Rounded cells overexpressing GLTP rarely migrated into the centre of the wound, and remained located at the wound edges or non-wound areas.

Next, we evaluated the impact of GLTP-induced rounding on cellular dynamic processes such as mobility and endocytosis. As shown in Figure 3A–C, normal-shaped cells were able to migrate long distances, as indicated by their frequent detection in the center of the wound in wound-scratch assays [21]. In contrast, rounded cells rarely migrated into the center of the wound (Fig. 3D). Instead, the rounded cells remained located at the wound edges or in non-wound areas.

**Figure 4:**
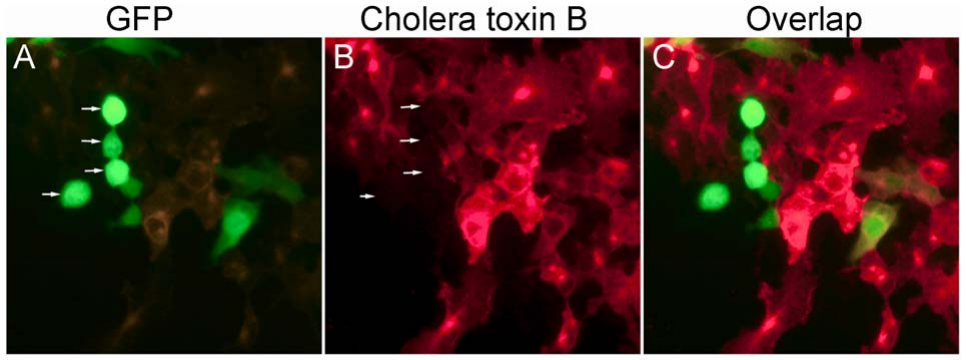
Rounded HeLa cells overexpressing GLTP are endocytotically-impaired. **A**) GFP-GLTP overexpression induces rounding of HeLa cells; **B**) **& C**) Cholera toxin B is rapidly internalized (within 30 min) by normal irregular-shaped HeLa cells, but not by rounded cells overexpressing GLTP.

Uptake of cholera toxin B (CTxB) was used to probe endocytosis by the rounded cells. Typically, cells can endocytose CTxB after it binds to the cell surface glycosphingolipid, GM1 [22], [23]. Figure 4 shows epifluorescence micrographs of HeLa cells labeled with Alexa555-CTxB. The fluorescent CTxb was rapidly internalized by normally-shaped control cells. However, in rounded cells overexpressing GLTP, uptake of CTxB did not occur.

### Does GLTP overexpression alter cell adhesion, viability, or apoptotic status?

**Figure 5:**
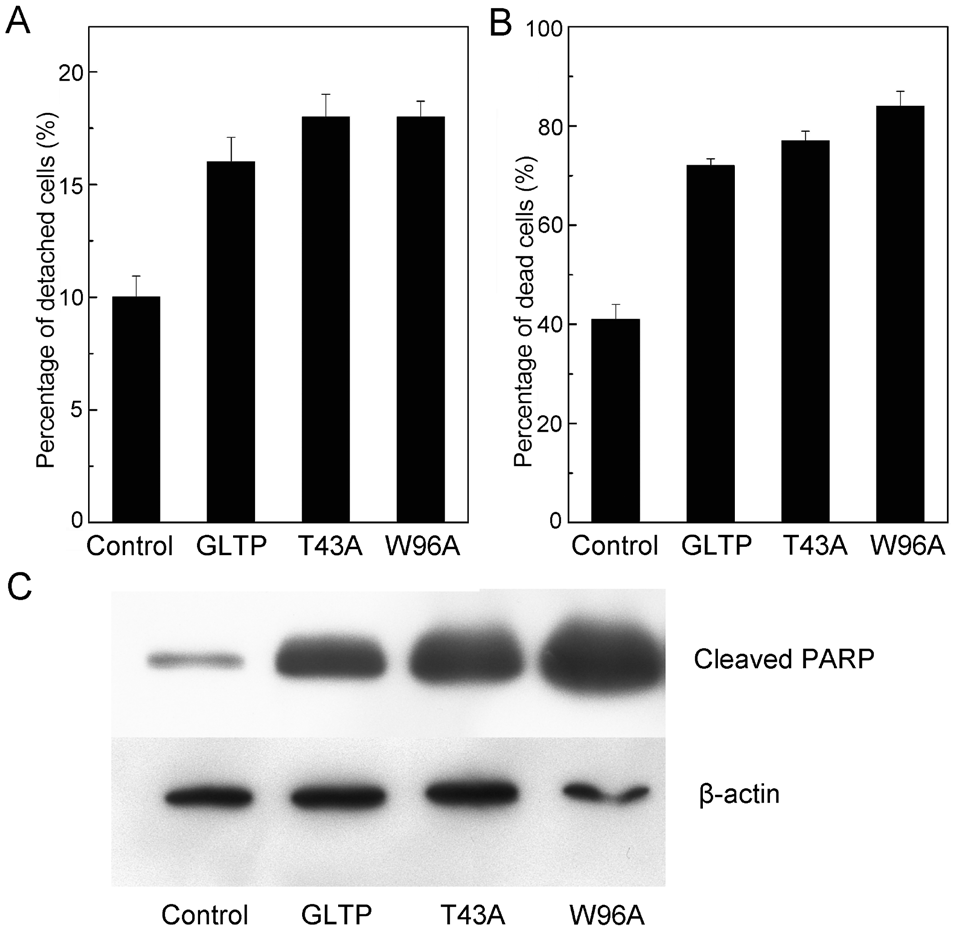
GLTP overexpression induces cell detachment and death. HeLa cell monolayers were transfected and incubated for 48 h with mock control, GLTP, GLTP-T43A, and GLTP-W96A plasmids. **A**) Elevated cell detachment in cells overexpressing GLTP, GLTP-T43A, or GLTP-W96A. Pelleting of cells floating in the media by centrifugation enabled counting of nonadherent cells; whereas, brief trypsination, washing and resuspension enabled counting of adherent cells. A minimum of ∼200 cells were counted from triplicate dishes to establish the percentages of detached cells (mean values+standard errors). **B**) Elevated cell death in nonadherent cells overexpressing GLTP, GLTP-T43A, or GLTP-W96A. Nonadherent cell viability was quantified by Trypan blue staining. **C**) Elevated apoptosis in nonadherent cells overexpressing GLTP, GLTP-T43A, or GLTP-W96A. Western immnunoblotting using antibody against cleaved PARP enabled assessment of apoptotic status. Beta-actin expression served as control.

**Figure 6:**
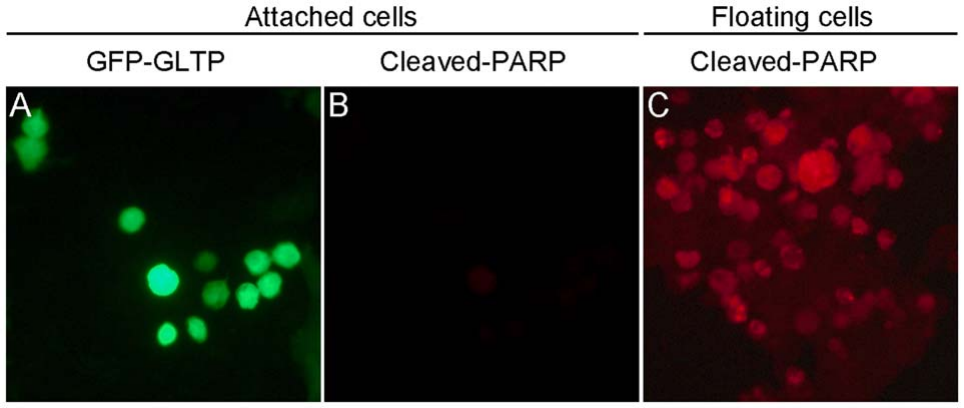
PARP cleavage in HeLa cells overexpressing GLTP remains low in adherent cells but is elevated in detached cells. **A**) Substratum-attached HeLa cells overexpressing GFP-GLTP show a rounded phenotype; **B**) Same field for A) but cells have been treated with antibody that detects cleaved PARP; **C**) Detached HeLa cells overexpressing GLTP stained with antibody that binds cleaved PARP.

The strong mitigation of cellular dynamics raised the issue of whether overexpression of GLTP significantly affects cellular adhesion and viability. To analyze for altered adhesion, cells attached to the dish (attached) or floating in the medium (nonadherent) were collected and counted. Figure 5A shows that detachment increased significantly when cells were overexpressing GLTP (∼16%) compared to mock-transfected cells (8–10%). Viability analyses by trypan blue staining revealed significantly elevated cell death in detached cells overexpressing GLTP (Fig. 5B). The increased cell death occurred regardless of whether a functional glycolipid binding site was present in GLTP. Further analysis of the nonadherent cells for apoptosis was performed by Western immunoblotting for poly(ADP-ribose) polymerase (PARP) cleavage, a classic indicator of caspase activation [24]. Figure 5C clearly shows dramatically elevated cleaved PARP in the detached cells overexpressing GLTP, consistent with anoikis, namely, detachment-related apoptosis. In contrast, rounded adherent cells overexpressing GLTP did not take up trypan blue (data not shown) and exhibited only slight elevation in cleaved PARP (Figs. 6A & 6B).

### Do other GLTP orthologs induce cell rounding?

**Figure 7:**
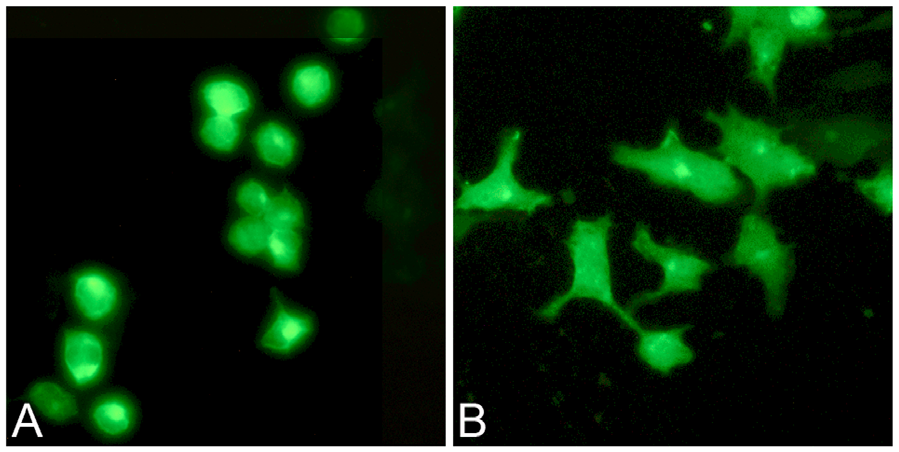
Cell morphological changes induced by overexpression of FAPP2 or ACD11. HeLa cells overexpressing GFP-FAPP2 (**A**) and GFP-ACD11 (**B**) were analyzed by fluorescence microscopy at 48 h after transfection.

We next determined whether the rounded cell phenotype could be induced by other GLTP-folds. The tested proteins included human FAPP2, which contains a GLTP-fold as its C-terminal domain that can transfer GSLs [17] as well as the plant GLTP ortholog, ACD11, which has moderate ability to transfer sphingomyelin [25]. As shown in Figure 7B, no change in HeLa cell morphology was induced by overexpression of ACD11. However, FAPP2 overexpression also induced cell rounding (Fig. 7A) that was morphologically similar to the rounding induced by wtGLTP overexpression.

### Can GLTP-induced cell rounding be regulated by interaction with other proteins?

To identify protein interaction partners capable of regulating the GLTP-induced changes in cell shape, yeast two-hybrid screening of a human brain cDNA library was performed. One positive clone was identified that encoded the C-terminal region of human delta(δ)-catenin (672–1225 a.a.) (Fig. S3). δ-catenin belongs to the p120-catenin (p120ctn) protein family characterized by ten Armadillo repeats that bind to cadherins. δ-catenin is typically expressed in neurons [26]–[28], but also has been observed in certain cancer cells [29]–[32] as well as in vascular endothelial cells during angiogenesis [33]. δ-catenin previously has been shown to promote dendritic outgrowths and cell spreading [34]–[38].

**Figure 8:**
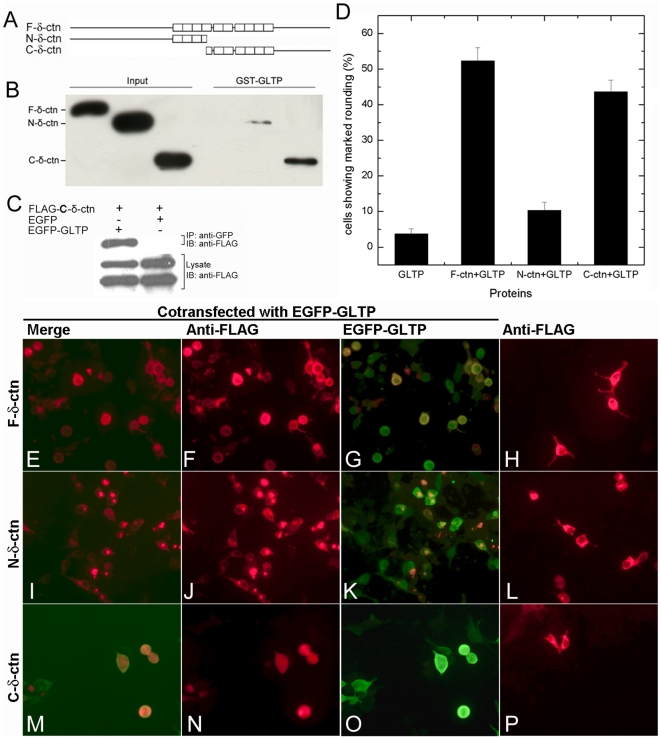
C-terminal region of δ-catenin interacts with GLTP and accelerates the phenotypic change induced by GLTP overexpression. **A**) Schematic of δ-catenin constructs. N-terminal δ-catenin = a.a. 1–693; C-terminal δ-catenin = a.a. 672–1225. **B**) GST pull-down assay and **C**) Co-immunoprecipitation assay indicate that the C-terminal region of δ-catenin interacts with GLTP. **D**) Percentages of cells showing marked rounding at 24 h after transfection. **E–P**) Epifluorescence analysis of changes in HeLa cell shape induced by GLTP and δ-catenin by 24 h. **H**) HeLa cells transfected with FLAG-δ-catenin (full length), **L**) FLAG-N-terminal-δ-catenin, or **P**) FLAG-C-terminal δ-catenin. HeLa cells cotransfected with GFP-GLTP and: FLAG-δ-catenin (full length) (**E, F, G**); FLAG-N-terminal-δ-catenin (**I, J, K**); or FLAG-C-terminal δ-catenin (**M, N, O**).

To independently verify interaction between GLTP and δ-catenin, we used affinity pull-down approaches to assess the binding of δ-catenin overexpressed in HEK 293T cells by GST-GLTP. As shown in Fig. 8B, GST-GLTP binds with the C-terminal region of δ-catenin (672–1225 a.a.), but not with the N-terminal half (1–692 a.a.) of δ-catenin. GST pull-down failed to show binding of GST-GLTP with full length δ-catenin, suggesting that the δ-catenin N-terminal region may regulate the interaction between GLTP and the C-terminal region of δ-catenin. The interaction between the C-terminal region of δ-catenin and GLTP also was confirmed using co-immunoprecipitation assays (Fig. 8C).

To determine the functional relevance of interaction between GLTP and C-terminal δ-catenin, we tested the effect of δ-catenin expression on GLTP in two ways. First, we examined whether coexpression of C-terminal δ-catenin along with GLTP altered glycolipid intermembrane transfer in HeLa cells (Fig. S4). However, in the recovered cytosolic fraction, glycolipid intervesicular transfer by GLTP was unaffected by C-terminal δ-catenin. Next, we examined the effect of coexpression of GFP-tagged GLTP and FLAG-tagged δ-catenin on cell morphology. As shown in Figure 8H, full length δ-catenin by itself induced dendritic-like protrusions in HeLa cells, consistent with previous observations in NIH 3T3 fibroblasts and in primary hippocampal neurons [34], [37]. Neither the N-terminal nor C-terminal region of δ-catenin by itself was able to induce the dendritic-like protrusions (Figs. 8L & 8P). Next, we cotransfected HeLa cells with GFP-tagged GLTP along with FLAG-tagged δ-catenin. Remarkably, no induction of dendritic-like protrusions occurred, but acceleration of cell rounding was evident (Fig. 8E–G). Notably, the accelerated cell rounding was observed during coexpression of GFP-GLTP with C-terminal δ-catenin (Fig. 8M–O), but not with N-terminal δ-catenin (Fig. 8D, I–K). In such cells, ∼50% displayed the rounded phenotype by 24 h compared to cells overexpressing only GFP-GLTP, which showed almost no rounding at 24 h (Fig. 1F) but significant rounding (∼70%) by 48 h (Fig. 1B, 1E, & 1F).

## Discussion

The present study provides the first evidence that elevated human GLTP expression can dramatically alter cell phenotype. The morphological change begins ∼24 h after transfection, namely, one doubling time for HeLa cells, and is manifested as a substantially rounded cell shape instead of the extended and elongated shape typical for HeLa cells. The GLTP-induced rounding of cells inhibits their mobility and ability to endocytose. Nonetheless, the vast majority of cells remain adherent and viable, showing little evidence of increased apoptosis. Induction of the round cell phenotype depends upon the presence of a functional glycolipid binding site in the GLTP fold. This finding is noteworthy because GSLs have long been known to play important roles in cell adhesion processes [1], [19], [20].

The phenotypic alterations induced by GLTP overexpression differ from those of epithelial-to-mesenchymal (EMT)-like transitions in important ways [39]. The latter are characterized by loss of cell-cell adhesion with cell shape becoming more extended and elongated, allowing for increased migration. In HEK-293 cells that have undergone EMT transition, the increased mobility is evident in both Transwell and wound-healing assays [40]. No such mobility increase was induced by GLTP overexpression. It also is noteworthy that HeLa, HEK-293, and A549 lung cells all can undergo EMT-like transitions [41]–[44]. In contrast, GLTP-induced cell rounding was observed in HeLa and HEK-293 cells but not in A549 cells. Thus, the cell rounding process induced by GLTP overexpression appears to differ from EMT-like transitions. Further studies will be needed for complete characterization including identification of associated molecular markers.

Exactly how overexpression of human GLTP or FAPP2 leads to the round cell phenotype remains to be elucidated but clearly is linked to the glycolipid binding/transferring ability of these GLTP-folds. Normally, the glycolipid transferring function of FAPP2 within the Golgi involves targeting by its N-terminal pleckstrin homology domain [10], [12], [17]; whereas, GLTP resides in the cytosol [45] but can interact with the endoplasmic reticulum via its FFAT-like motif [46] and deliver glucosylceramide to the plasma membrane [18]. Typically, the simplest GSLs (e.g., glucosyl- and galactosylceramides) are cytosolically exposed and directly accessible to GLTP-folds [6], [8], [11], [18]. Such localization of excessive glycolipid binding GLTP-folds could impact cellular processes involving more complex GSLs by homeostatic disruption of GSL metabolism. We speculate that abnormally large amounts of GLTP or FAPP2 in the cytosol could limit availability of the simple glucosylceramide (GlcCer) produced by the cytosol-facing GlcCer synthase and needed for generation of higher GSLs involved in cell-cell interactions on the cell surface. The net effect could be reduced levels of complex GSLs that participate in cellular adhesive interactions [1], [2], [19], [20]. Of course, the preceding ideas will need to be evaluated experimentally.

We also discovered that GLTP-induced cell rounding is accelerated by overexpression of δ-catenin, a member of the p120-catenin subfamily of armadillo proteins. The function(s) of p120 protein family members are targeted to membrane junctional regions where cell-cell interactions occur and include stabilization of cadherins by binding to a highly conserved sequence in the juxtamembrane region [26]–[28]. In neuronal cells, δ-catenin is localized to the postsynaptic adherens junction where, along with Rho GTPases, it induces dendritic protrusions and regulates neurite elongation and branching [34]–[38]. δ-Catenin expression also has been observed in prostate cancer cells [29]–[32] and in vascular endothelium where it plays a role in regulating endothelial cell mobility and angiogenesis in tumors and wound healing [33]. Given the ability of δ-catenin to impact cell adhesion interactions that control cell mobility and contacts with adjacent cells, it is not surprising that this p120 catenin member interacts with E-cadherin, S-SCAM, p0071, Densin-180, PSD-95, Abl, Cortactin, sphingosine kinase, and Kaiso [27], [28]. Our finding of δ-catenin interaction with GLTP was unexpected because GLTP expression had never previously been linked to cellular adhesive interactions. What is especially remarkable about this finding is the resulting mitigation of δ-catenin-induced dendritic branching and accelerated transition to the round cell phenotype that occurs during coexpression of GLTP and δ-catenin. While the molecular details remain to be mapped and fully elucidated, what is clear is that the C-terminal region of δ-catenin is of primary importance in its functional interaction with GLTP. Taken together, the findings reported herein show for the first time that GLTP overexpression can markedly affect cell phenotype and suggest that strict regulation of GLTP expression is needed to avoid disruptions in the production of complex GSLs known to play roles in maintaining healthy and viable cells-cell interactions.

## Materials and Methods

The open reading frames (ORFs) for human GLTP (627 bp) and δ-catenin [full length (3675 bp), N-terminal region (2076 bp), or C-terminal region (1662 bp)] were cloned into pEGFP (Clontech) or pFLAG-CMV4 (Sigma) vector, respectively. GLTP point mutants were created by QuikChange mutagenesis (Stratagene). GLTP-siRNAs were produced using pSuper.retro.puro vector (gift of Drs. Albert Bendelac & Yuval Sagiv), which we modified further by inserting the enhanced green fluorescent protein ORF at the C-terminus of the puromycin N-acetyl transferase coding sequence (pSuper.puro-egfp). Short hairpin RNAs (shRNA) against human GLTP were designed using an on-line siRNA design program (Ambion) and subcloned into the BglII/XhoI sites of pSuper.puro-egfp. HeLa and HEK293-T cells (American Type Culture Collection) were maintained in DMEM supplemented with 10% fetal calf serum and penicillin/streptomycin under 5% CO_2_ at 37°C. Cells were transfected using Expressfect™ (Denville Scientific Inc). SDS-PAGE/Western immunoblots were analyzed using anti-GFP (Santa Cruz Biotechnoloy), anti-β-actin (Delta Biolabs), or anti-FLAG (Sigma) antibody.

### Immunocytochemistry

HeLa cells grown on glass coverslips were fixed in 4% paraformaldehyde for 20 min at room temperature, rinsed with PBS, and permeabilized with 0.25% Triton X-100 in PBS for 10 min. After blocking for 1 h with 1% BSA in PBS containing 0.1% Tween 20, cells were incubated with polyclonal anti-FLAG antibody (Sigma; 1∶500) in PBS with 1% BSA for 2 h at room temperature and then incubated with Rodamine Red X-conjugated-goat anti-mouse (Jackson Laboratories; 1∶200) in PBS for 1 h at room temperature. After several rinses in PBS, coverslips were mounted with Prolong Gold antifade reagent (Invitrogen) and cells were examined using a Leica DM IRB fluorescence microscope or a Nikon confocal microscope.

### Endocytosis of cholera toxin B (CTxB)

HeLa cells were washed two times with PBS, incubated with Alexa Fluor 555-conjugated CTxB (10 µg/ml, Invitrogen) for 30 min at 4°C, and then warmed to 37°C for 30 min to allow the endocytosis of CTxB. Cells were then washed three times with PBS, fixed in 4% paraformaldehyde, and examined by epifluorescence microscopy.

### Wound closure assays

HeLa cells in DMEM with 10% FCS were transfected with GLTP and incubated for 24 h at 37°C. The confluent cells were wounded with a linear scratch by a sterile pipette tip. After washing, cells were incubated for another 24 h at 37°C and the wounded cell monolayers were analyzed by epifluorescence microscopy.

### Yeast two hybrid analyses

Yeast two hybrid screening was performed using the Matchmaker GAL4 Two-Hybrid System 3 (Clontech). The yeast strain AH109, pretransformed with pGBKT7-*GLTP* ORF, was mated with MATα yeast strain Y187 cells containing a human brain cDNA library cloned into pACT2, a vector containing the GAL4 DNA binding domain. Diploid yeast cells were first selected for growth on SD-glucose plates lacking tryptophan (−Trp), leucine (−Leu), and histidine for 13 days at 30°C. Plasmid DNAs were isolated from colonies that activated all three yeast reporter genes (HIS3, ADE2, lacZ) using Yeast DNA Isolation kit (Stratagene). To confirm the mating result, the AH109 yeast cells were cotransformed with empty pGBKT7 vector or pGBKT7 containing GLTP ORF and pACT2 vector containing C-terminal catenin. Transformed cells were tested for growth and development of blue color on SD plates supplemented with X-α-gal, but lacking tryptophan, leucine, histidine, and adenine.

### GST pull-down assays

GST fusion proteins were expressed in E. coli BL21 strain and affinity-purified using glutathione-Sepharose 4B beads (Amersham Biosciences). To express FLAG or EGFP fusion proteins, pFLAG-CMV4 or pEGFP plasmids containing δ-catenin ORFs (full length, N-terminal (1–692 a.a.) or C-terminal (672–1225 a.a.) were used to transfect HEK293 T cells. Cell lysate, prepared 48 h after transfection, was mixed with the GST fusion proteins in binding buffer (500 µl; 50 mM Tris, 150 mM NaCl, 0.25% NP40) for 5 h at 4°C. Agitation was followed after addition of 50% glutathione-Sepharose bead slurry (30 µl). The beads were then washed five times with binding buffer (800 µl). Bound proteins were eluted by washing with SDS gel loading buffer and boiled for 5 min. After SDS-PAGE (8% gels), Western immunoblotting was performed with mouse anti-FLAG or anti-GFP antibody.

### In vivo coimmunoprecipitation pull-down assays

HEK293 T cells were cotransfected with pFLAG- δ-catenin and pEGFP-GLTP or pEGFP empty vector using TransIT-LT1 reagent (Mirus Inc.). At 48 h post transfection, cell lysates were prepared and incubated overnight with anti-GFP monoclonal antibody. The resulting complexes were collected by immunosorbing to Protein A & G Plus agarose beads (Santa Cruz) for 5 h at 4°C. Beads were washed 5 times with lysis buffer (800 µl; 50 mM Tris, 150 mM NaCl, 0.5% NP; (Mammalian Cell-PE LB™; G-Biosciences). Protein complexes were eluted using SDS gel loading buffer, separated by SDS-PAGE (8% gels), and analyzed by Western immunoblotting using anti-FLAG antibody.

## Supporting Information

Figure S1 Human GLTP structure and locations of point mutations. Structure (PDB 1SX6) was solved previously by X-ray crystallography [13]. T43A and W96A point mutation locations are shown with respect to bound glycolipid, lactosylceramide (yellow).
(PDF)

Figure S2 GLTP overexpression induces changes in cell morphology in HEK-293 cells but not in A549 lung cells.
(PDF)

Figure S3 Yeast two-hydrid identification of δ-catenin as GLTP interaction partner. AH109 yeast cells were transformed with pGBKT7-GLTP (plate 1), pACT2-C-terminal δ-catenin (plate 3), cotransformed with pGBKT7-p53 and pTD1-1 (plate 2), or with pGBKT7-GLTP and pACT2-C-terminal δ-catenin (plate 4). Transformants were selected for growth on SD-glucose media supplemented with X-α-Gal, but lacking histidine, adenine, leucine and tryptophan.
(PDF)

Figure S4 Effect of δ-catenin on GLTP transfer activity. Intermembrane transfer of radiolabeled GlcCer by HeLa cell cytosol transfected with GFP (control) or with GFP-GLTP, or cotransfected with GFP-GLTP and FLAG-δ-catenin (full length, N-terminal region, or C-terminal region).
(PDF)
