# Supplementary figures and images for: Human Glycolipid Transfer Protein (GLTP) Expression Modulates Cell Shape
Source: PLoS One. 2011 May 18;6(5):e19990. doi: 10.1371/journal.pone.0019990 (PMC3097243; doi:10.1371/journal.pone.0019990)

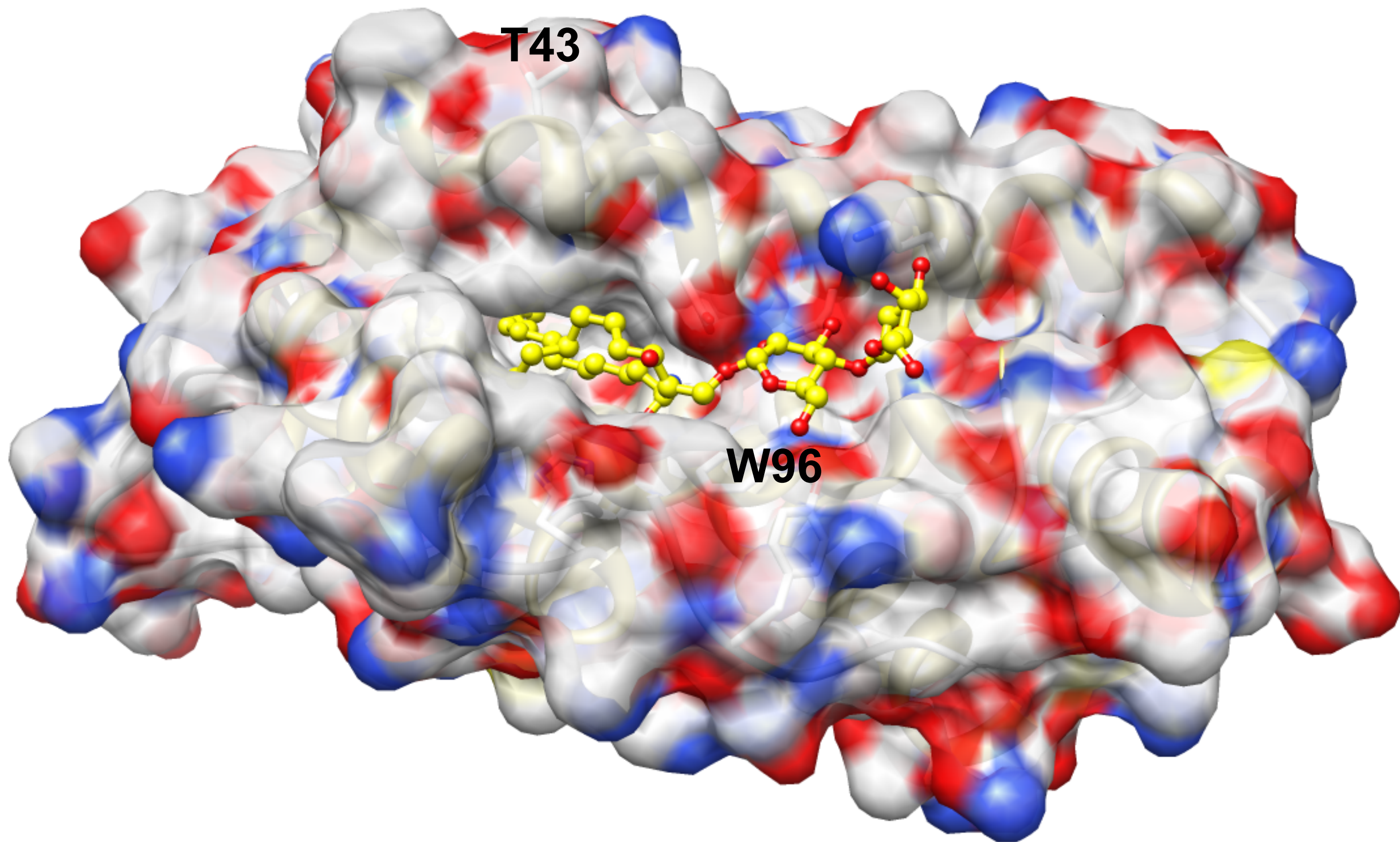

Supplement: Figure S1 — Human GLTP structure and locations of point mutations. Structure (PDB 1SX6) was solved previously by X-ray crystallography [13]. T43A and W96A point mutation locations are shown with respect to bound glycolipid, lactosylceramide (yellow). (PDF) [file pone.0019990.s001.pdf]

HEK-293

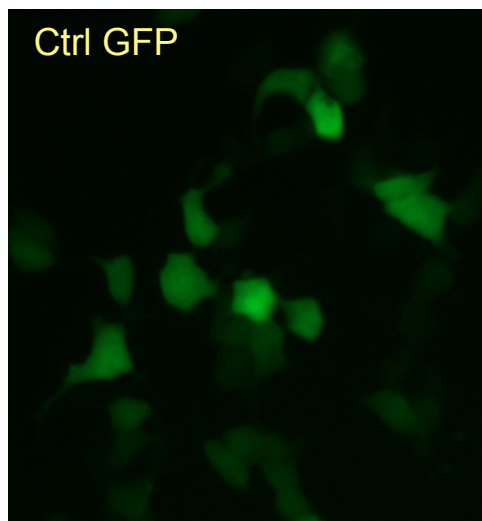

A549

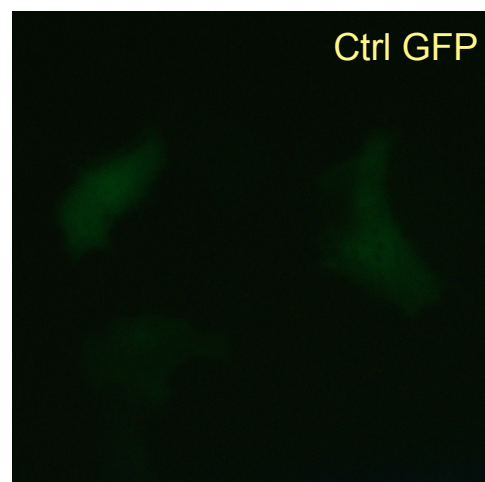

24 h

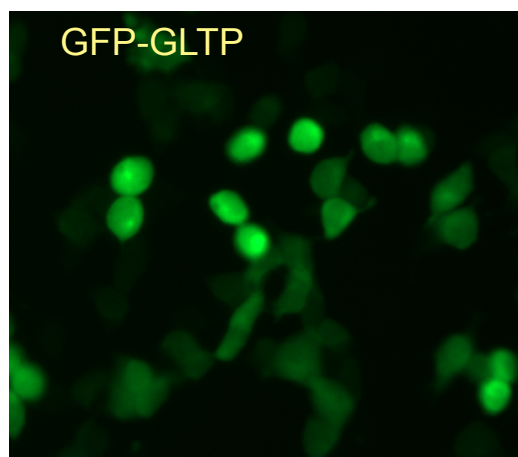

24 h

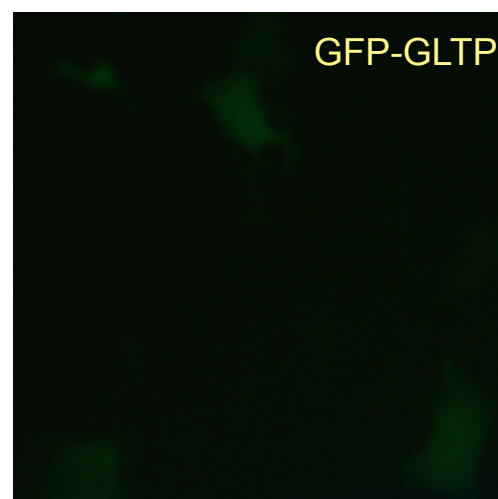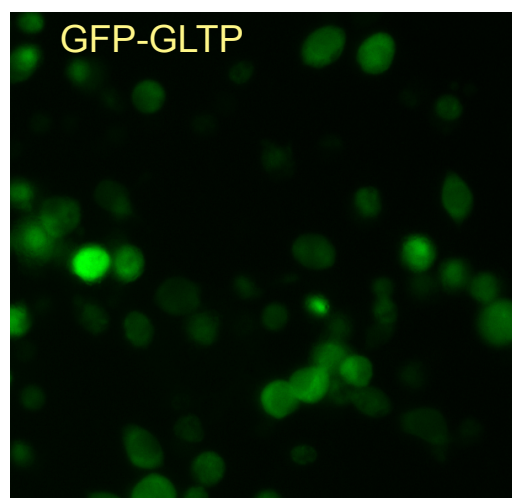

72 h

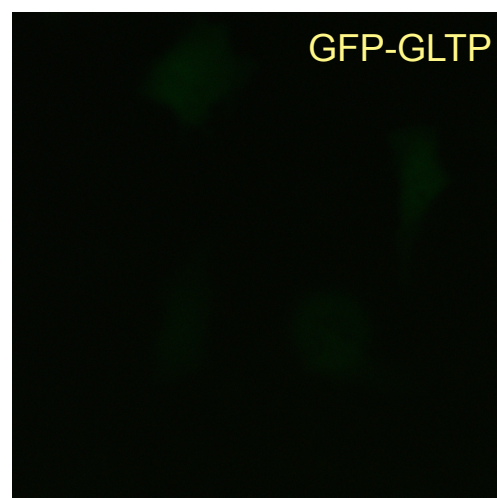

Supplement: Figure S2 — GLTP overexpression induces changes in cell morphology in HEK-293 cells but not in A549 lung cells. (PDF) [file pone.0019990.s002.pdf]

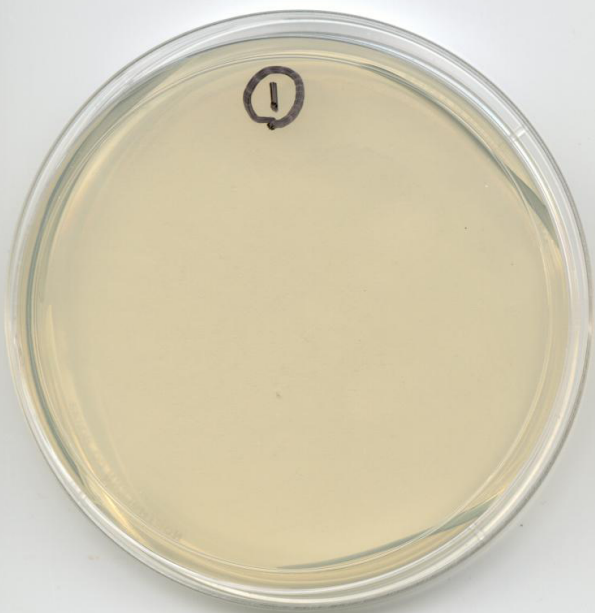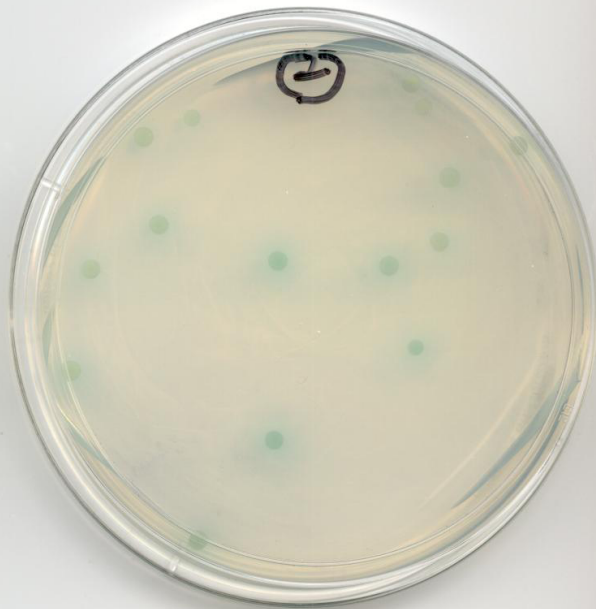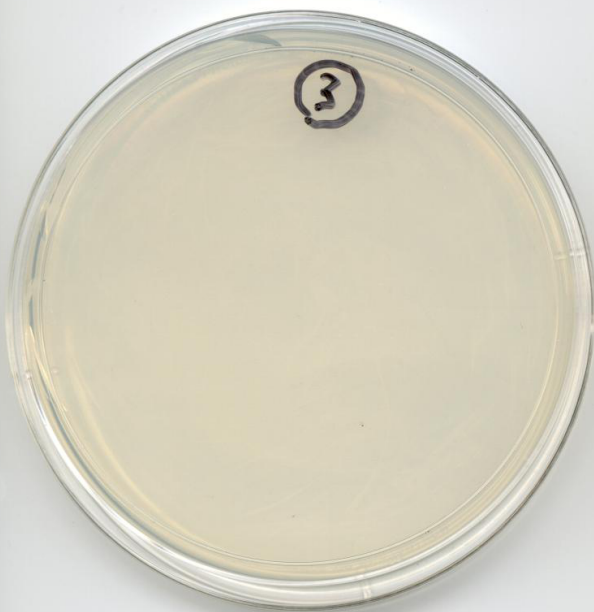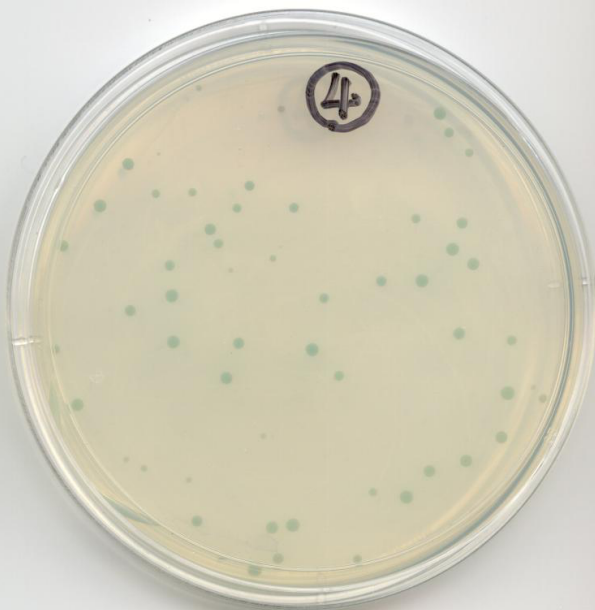

Supplement: Figure S3 — Yeast two-hydrid identification of δ-catenin as GLTP interaction partner. AH109 yeast cells were transformed with pGBKT7-GLTP (plate 1), pACT2-C-terminal δ-catenin (plate 3), cotransformed with pGBKT7-p53 and pTD1-1 (plate 2), or with pGBKT7-GLTP and pACT2-C-terminal δ-catenin (plate 4). Transformants were selected for growth on SD-glucose media supplemented with X-α-Gal, but lacking histidine, adenine, leucine and tryptophan. (PDF) [file pone.0019990.s003.pdf]

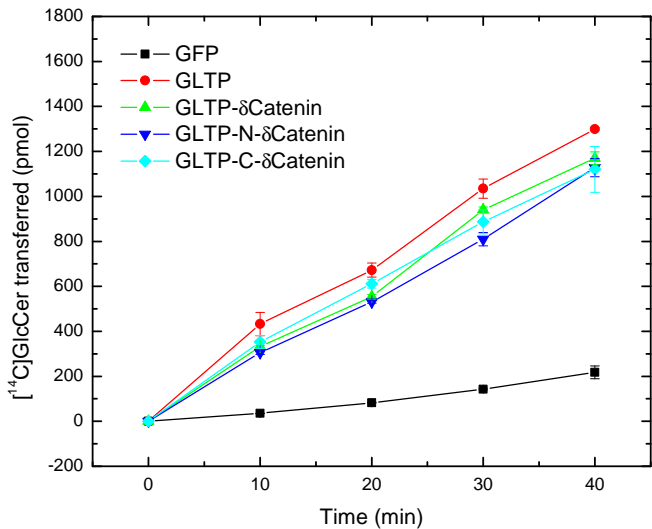

Supplement: Figure S4 — Effect of δ-catenin on GLTP transfer activity. Intermembrane transfer of radiolabeled GlcCer by HeLa cell cytosol transfected with GFP (control) or with GFP-GLTP, or cotransfected with GFP-GLTP and FLAG-δ-catenin (full length, N-terminal region, or C-terminal region). (PDF) [file pone.0019990.s004.pdf]
